# Population structure, selection signal and introgression of gamecocks revealed by whole genome sequencing

**DOI:** 10.1186/s40104-025-01154-4

**Published:** 2025-02-08

**Authors:** Naiyi Xu, Linyun Zhang, Feifan Chen, Zhengfu Feng, Jiangtao Zheng, DongHua Li, Yongju Zhao, Xiangtao Kang

**Affiliations:** 1 College of Animal Science and Technology, Southwest University, Chongqing, 400715 China; 2 Chongqing Key Laboratory of Herbivore Science, Chongqing, 400715 China; 3 College of Animal Science and Technology, Henan Agricultural University, Zhengzhou, 450046 China

**Keywords:** Gamecock, Genomic introgression, Population structure, Selection signal, Whole genome re-sequence

## Abstract

**Background:**

As an important genetic resource of chickens, gamecock has unique morphological and behavioral characteristics such as large size, muscular development and strong aggression, making it a good model for studying muscle development and behavior patterns, as well as an excellent breeding material. Gamecocks are distributed worldwide, forming different breeds and strains. However, the single or multiple origin of global gamecocks has not been fully established and much remains unknown about genetic introgression events between gamecocks and other chickens. Therefore, in this study, based on whole genome data of gamecocks, Chinese indigenous chickens, commercial chickens and wild jungle fowls, we performed population structure analysis, selection signal analysis, and genomic introgression analysis of gamecocks.

**Results:**

The population structure analysis revealed that gamecocks have multiple origins. In addition, we used *F*st, π-Ratio and XP-EHH methods to explore the candidate signatures of positive selection in gamecocks. A total number of fifteen shared candidate genes were identified using the three different detection strategies. Among these candidate genes, *ETV1*, *DGKB*, *AGMO*, *MEOX2*, *ISPD* and *PLCB4* are related to the growth and muscle development, fighting performance and neurodevelopment of gamecocks. Furthermore, we detected the introgression event at the *MYBPHL* region from the *Gallus sonneratii* into Euramerican gamecocks and at the *CPZ* gene region from the *Gallus gallus gallus* into multiple gamecock populations, respectively, indicating the introgression from the wild junglefowl may impact the skeletal and muscle development as well as aggressive behavior of gamecocks.

**Conclusions:**

In summary, we conducted a genome-wide exploration of gamecocks from multiple regions worldwide. Our analysis confirmed multiple origins of gamecocks and detected several candidate genes that are possibly related to important traits and characteristics in gamecocks. More importantly, this is the first study that has detected introgression events and genes from wild jungle fowls to gamecocks, which provides evidence of the wild jungle fowls contributing to the genetic diversity of gamecocks. Our findings offer new perspectives on the impact of introgression on gamecocks, and provide a basis for further resource conservation and utilization of gamecock, as well as provide excellent material for the genetic improvement of domestic chickens.

**Supplementary Information:**

The online version contains supplementary material available at 10.1186/s40104-025-01154-4.

## Background

The domestic chicken, as the most abundant and widely distributed terrestrial vertebrates, with a population size of more than 33 billion and production of more than 25 billion chickens per year [1], is an important source of excellent and low-cost animal protein for human and plays an important role in the national economy. It is generally accepted that the red jungle fowl (RJF) subspecies *Gallus gallus spadiceus* from southwestern China, northern Thailand and Myanmar are the wild progenitor of domestic chickens [2]. According to the utility of domestic chicken for humans, they are generally divided into several categories, such as egg and meat consumption, entertainment, cultural, religious and ornamental. Gamecocks have been used specifically for cockfighting through long-term selection, which are spread distinct geographical areas all over the world.

There are a lot of gamecock breeds distributed in different countries around the world. Chinese gamecock breeds are generally classified as Henan (HN), Luxi (LX), Turpan (Tur), Nanjiang (NJ), Xishuangbanna (XSBN), Yunnan (YN) and Wanbei (WB) gamecock. Oshamo is main Japanese (JPN) gamecock variety. Besides, there are Southeast Asia (SEA), Europe (EUR), North America (NA), and South America (SA) gamecock breeds, such as Sumatran gamecock, Spanish gamecock, Mexican gamecock, and Peruvian gamecock. The domestication origin of gamecocks has not been fully established [3–5]. Although different gamecock breeds have different characteristics, long and severe selection has led to the accumulation of some unique and shared morphological and behavioral traits, such as muscular and robust bodies and extremely aggressive [6, 7]. The lack of a systematic breeding system, the small scale of feeding and the ease of crossbreeding may lead to genetic admixture between gamecock and indigenous, commercial (C) chicken and wild jungle fowl [3, 8, 9]. What’s more, the origin of gamecock is not well clear, and genetic introgression between gamecocks and other chickens, and the effect of introgression on the phenotype of the gamecocks are also not well understood.

Natural and artificial selection as important driving forces during domestication, breed formation and adaptation leave genetic “imprint” in specific regions of the genome, such as increased allele frequencies, reduced genetic polymorphism, extensive linkage disequilibrium and long-range extended haplotype homozygosity [10]. Population genetic analyses can help elucidate the origins of breeds, population genetic structure, patterns of genomic variation, selective signatures, thus promoting genetic resources conservation and breeding programs. Advanced molecular tools and sequencing methods have greatly facilitated population genetics research, such as whole genome sequencing, which has been widely applied in studies of various domesticated animals [11–14]. At present, there are some studies on Chinese gamecock and other foreign gamecocks respectively [3, 5–9], while, there are fewer comprehensive studies on almost all gamecocks around the world. In subsequent whole genome sequencing studies, incorporating more gamecock breeds and taking the C chicken and Chinese indigenous chickens into account could help us fully and comprehensively uncover key variants/genes underlying signatures in gamecock chickens.

In this research, we re-sequenced and collected 231 whole genome of chicken individuals (Additional file 1: Table S1) [2, 4, 15–17], to explore the origin of global gamecocks, genetic introgression between gamecock and other chicken breeds, as well as pivotal candidate variants/genes underlying unique traits in gamecock chickens. This allow us to get a deeper and more comprehensive understanding of gamecock chickens, and thus better conserve and utilize the excellent breeding materials of gamecocks.

## Methods

### Sampling, sequencing and variant calling

We sampled 13 blood samples of HN gamecocks from Henan Province, China (Additional file 1: Table S1). Genomic DNA was extracted from blood samples of each individual using the standard phenol–chloroform method. Paired-end libraries with insert size of 300–500 bp were constructed for each individual using BGI Optimal DNA Library Prep Kit (BGI, China) following the manufacturer’s protocol, and then, the libraries were sequenced on the MGISEQ-2000 and 150 bp paired-end reads were generated. In addition, we collected the genome data of 218 chicken individuals across the world from public database, including 65 gamecocks from China (LX, Tur, NJ, XSBN, YN and WB), 37 gamecocks from other countries/regions (JPN, SEA, EUR, NA and SA), 35 Chinese indigenous chickens [Gushi (GS), Hetian (HT), Nixi (NX), Shouguang (SG)], 23 C, 58 wild jungle fowls representatives of all four species of wild jungle fowls (green jungle fowl (*G. varius*), Ceylon jungle fowl (*G. lafayettii*), grey jungle fowl (*G. sonneratii*) and RJF) and each of the five subspecies (*G. g. bankiva*, *G. g. gallus*, *G. g. jabouillei*, *G. g. murgha*, *G. g. spadiceus*) of RJF (Additional file 1: Table S1) [2, 4, 15–17].

All genome data we obtained were used to call the single nucleotide polymorphisms (SNPs). First, we removed low-quality bases and artifact sequences of raw data using Trimmomatic [18], and all clean reads were mapped to the chicken reference genome GRCg7b by Burrows-Wheeler Aligner “BWA-MEM” algorithm with default parameters [19]. Then, SAMtools [20] were used to sort bam files, “MarkDuplicates” of Picard (http://broadinstitute.github.io/picard) were used to identify potential Polymerase Chain Reaction duplicates, and “HaplotypeCaller”, “GenotypeGVCFs”, and “SelectVariants” of the Genome Analysis Toolkit (GATK) [21] were used to call and select candidate SNPs, respectively. Last, we used the “VariantFiltration” module of GATK to filter possible false-positive calls with the standard parameters as below: Variants by quality depth < 2; Fisher strand > 60; mapping quality rank sum test < −12.5; read position rank sum test < −8; mapping quality < 40.0; strand odds ratio > 3.0; the mean sequencing depth of variants (for all individuals) < 1/3 × and > 3 × ; maximum missing rate < 0.1; and SNPs were restricted to the two alleles. The remaining SNPs were annotated using Annovar. Imputation and phasing of SNPs were simultaneously performed using BEAGLE [22] with default setting.

### Population structure and mixture

The genetic relationships among gamecock populations were performed by NJ tree, principal component analysis (PCA), and ADMIXTURE. The NJ tree was constructed with PLINK based on the matrix of pairwise genetic distances from the autosomal SNPs data of 173 domestic chickens and 1 *G. varius* (Additional file 1: Table S1) and visualized by iTOL [23]. For PCA and ADMIXTURE, the autosomal SNPs of 173 domestic chickens (Additional file 1: Table S1) were filtered by VCFtools [24] with MAF (–maf 0.05), and LD-based pruning for the genotype data were performed by PLINK [25] with extended parameters (–indep-pairwise 50 5 0.2). The PCA was carried out using SmartPCA in the package EIGENSOFT [26] and the significance level of the eigenvectors was detected by the Tracy-Widom test. ADMIXTURE [27] was used to estimate the population genetic structure and run for each assumed genetic cluster (*K* = 2 to 10), where *K* was the assumed number of ancestries. The qp3Pop program in the ADMIXTOOLS package was used to calculate outgroup *f*3 statistics [28].

### Selection signal analysis

We screened genomic regions under selection of gamecock population by population differentiation index (*F*st) [29], the largest differences in genetic diversity (π-Ratio), and cross-population extended haplotype homozygosity (XP-EHH) [30, 31]. In the *F*st, π-Ratio, and XP-EHH tests, all the gamecock population (HN, LX, Tur, NJ, XSBN, YN, WB, JPN, SEA, EUR, NA and SA) as the object population and the non-gamecock population (C, GS, HT, NX, SG) as a reference population (Additional file 1: Table S1). We performed *F*st, π-Ratio using VCFtools [24] with the default settings, and employed XP-EHH analysis by selscan [32] with the default parameter. The *F*st, π-Ratio, and average normalized XP-EHH score were calculated for 40 kb sliding windows with 20 kb steps, and the top 1% windows were identified as candidate selective regions. The genes annotated in candidate selective regions were considered as candidate genes. The combination of multiple selection analysis methods can avoid bias and improve the power of identifying selective signatures. Therefore, we mainly focus on the candidate genes identified by all the above three tests. To better understand the biological functions and involved signaling pathways of candidate genes, GO and KEGG pathway enrichment analyses were performed using KOBAS [33], and corrected *P*-value < 0.05 were considered statistically significant for enrichment.

### Genomic introgression analysis

Genomic introgression analysis was conducted by autosomal SNPs data of 231 chickens (173 domestic chickens and 58 wild jungle fowls). Treemix [34] was used to determine possible gene flow between gamecocks and wild jungle fowls (Additional file 1: Table S1). We ran Treemix with migration events ranging from 0 to 9 (m = 0–9) and generated their corresponding residual matrices. The *G. varius* was specified as an outgroup in Treemix analysis. The *D*-statistic, also known as the ABBA-BABA test, was also implemented with Dsuite [35] to detect gene flow between gamecocks and other chicken population. The tree topology is as follows: P1 is C, P2 is gamecock and Chinese indigenous chicken, P3 is *G. sonneratii* or RJF, and O is *G. varius*. “Dtrios” module of Dsuite was used to calculate *D-*statistics for all possible trios of the above populations. Then, “Dinvestigate” module of Dsuite was used to evaluate the introgression level and locate the introgression region of the whole genome through a sliding window contained 100 informative SNPs with a step of 50 SNPs. The windows in the top 1% of *D* values were considered as the candidate introgression region, the genes of the introgression region annotated by the Biomart module of Ensembl (http://www.ensembl.org/biomart/martview) were considered as candidate introgression genes. The maximum likelihood (ML) tree were constructed for the introgression genes by IQ-TREE [36] and visualized with online iTOL [23]. We also compared the mean pairwise sequence divergence (dxy) between the introgressed gamecock population and wild jungle fowl using a sliding window with 50 kb sliding window and 10 kb step [37].

## Results

### Sequencing and genomic variant

A total of 13 HN gamecocks were selected for whole genome sequencing with an average depth of 11.10 × , and 218 chicken genomic sequences were obtained from publicly available data (Additional file 1: Table S1). The average mapping rate of the reads sequenced in this study was 99.42%, and the sequencing coverage was approximately 10.95 × per individual. In total, 17,563,493 SNPs were identified. Functional annotation of the polymorphic sites showed that the vast majority of SNPs were present either intergenic regions (52%) or intronic regions (28%). Besides, exons contained 1.6% of the total SNPs with 92,232 non-synonymous SNPs and 178,746 synonymous SNPs.

### Population genetic structure and relationships

**Fig. 1:**
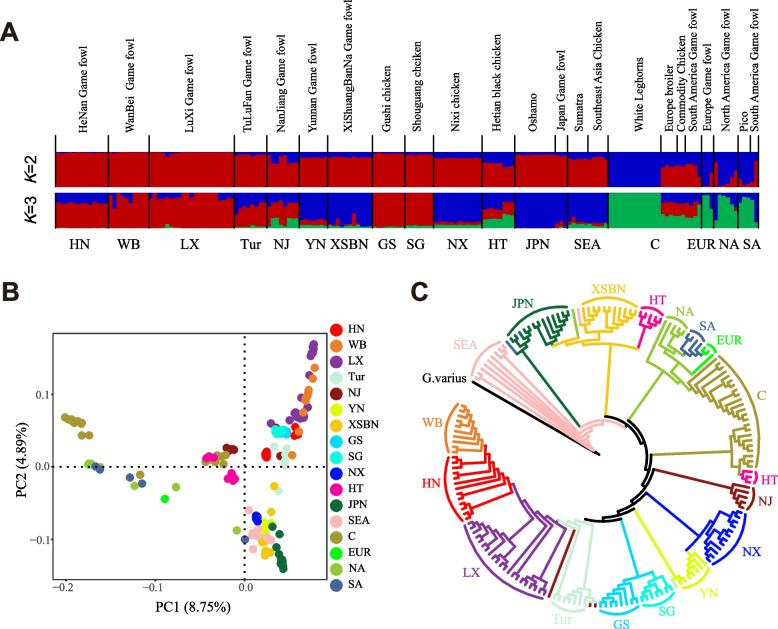
Population structuring and relationship among gamecocks and other chickens. **A** Genetic structure of gamecocks from different regions and other chicken breeds using ADMIXTURE program with *K* = 2 and *K* = 3. **B** PCA showing the first principal component (PC1) against the second principal component (PC2) of different chicken populations. **C** NJ tree constructed using whole genomic autosomal SNPs data. Each breed was labelled with different colors and abbreviation of breed name

To explore genetic structure and relationships among gamecocks from different regions and other chicken breeds, we conducted ADMIXTURE, PCA and NJ tree using whole genomic autosomal SNPs data. These analyses revealed clear geographic patterns among different chicken populations. In the ADMIXTURE analysis (Fig. 1A), we calculated the population admixture proportions by assuming *K* from 2 to 10. At *K* = 2, Asian chickens, including gamecocks and other Chinese indigenous chickens, are genetically distinct from European and American samples. At *K* = 3, Asian chickens further split into two subgroups: most Chinese gamecocks and domestic chickens, JPN, SEA, YN, XSBN gamecocks and NX chickens. Similarly, the genetic relationships are also supported by PCA (Fig. 1B), with most Chinese chicken populations forming their clusters, JPN, SEA, YN, XSBN and NX chickens forming one cluster, and European and American belong to one cluster. Expectedly, the result of the NJ tree (Fig. 1C) and outgroup *f*3 (Additional file 2: Fig. S1) is generally consistent with these findings.

### Genome-wide selective sweep analysis

**Fig. 2:**
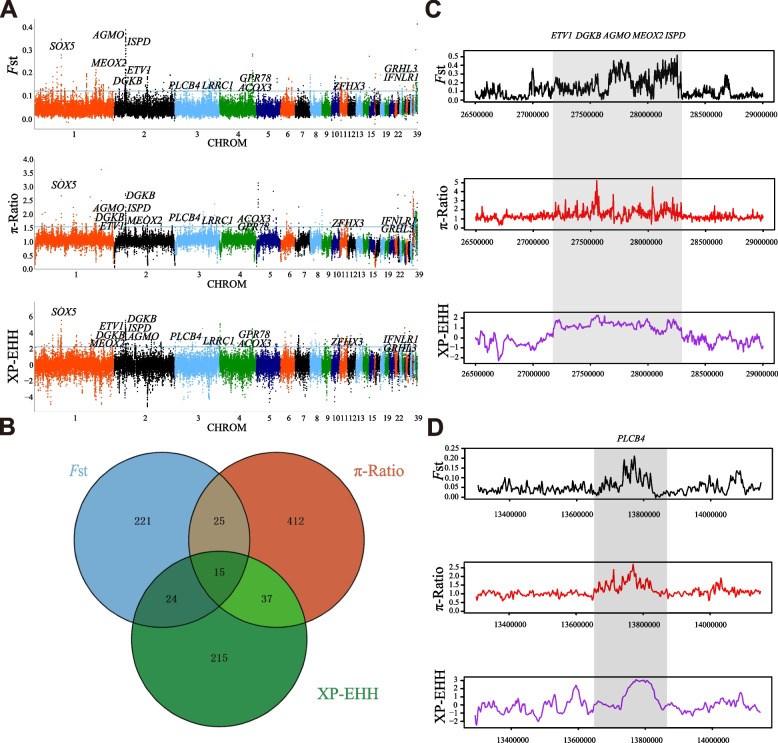
Genome-wide selective sweep analysis of gamecocks. **A** Manhattan plot of the *F*st, π-Ratio and XP-EHH values (y-axis) in windows of 40 kilobases (kb) using a 20 kb slide across all autosomes (x-axis). The blue horizontal line indicates the 99th percentile cutoff (top 1%) for extracting outlier. Candidate genes were identified by the three methods of *F*st, π-Ratio and XP-EHH are shown. **B** Venn diagram showing the candidate genes overlap among *F*st, π-Ratio and XP-EHH. **C** *F*st, π-Ratio and XP-EHH tests of *ETV1*, *DGKB*, *AGMO*, *MEOX2* and *ISPD* gene regions in gamecocks. **D** *F*st, π-Ratio and XP-EHH tests of the *PLCB4* genomic region

To identify candidate selective regions and genes within gamecock populations, we combined data from all gamecock populations and compared them with the C chickens and Chinese indigenous chickens using *F*st, π-ratio and XP-EHH approaches (Fig. 2A). In the three analyses, the outlier signals (top 1%) in the selective regions were identified as potential candidate regions, and genes annotated in these candidate regions were considered as potential candidate genes. Totally, 285, 489 and 291 potential candidate genes were identified from *F*st, π-ratio and XP-EHH, respectively (Fig. 2A, Additional file 1: Table S2–4). 949 unique candidate genes were found using the three methods (*F*st, π-ratio and XP-EHH), with fifteen candidate genes shared by all three methods (Fig. 2B, Additional file 1: Table S5). Besides, we performed functional enrichment analysis using KEGG pathways and GO terms for the three lists of potential candidate genes. Over-representation analysis (corrected *P*-value < 0.05) of GO terms shows that gamecock has increased GO categories related to “receptor-mediated endocytosis”, “integral component of plasma membrane”, “keratin filament”, “sarcomere”, “modulation of chemical synaptic transmission”, “glutamatergic synapse”, “proton-transporting ATP synthase activity”, and “rotational mechanism” (Additional file 1: Table S6–8). In KEGG pathways analysis, candidate genes in gamecock population are significantly over-represented (corrected *P*-value < 0.05) in “NOD-like receptor signaling pathway”, “mitophagy-animal pathway”, “metabolic pathways”, and “Glycosaminoglycan biosynthesis-heparan sulfate/heparin pathway” (Additional file 1: Table S9–11).

Fifteen candidate genes were identified using multiple approaches, showing strong selection signatures in gamecock populations. In particular, a strong selective sweep region spans around 1 Mb region (27,175,233–28,296,806 bp) on chromosome 1 exhibiting high *F*st, π-Ratio, and XP-EHH values (Fig. 2C). This region contains five candidate genes associated with various biological functions: ETS variant 1 (*ETV1*) related to motor-neuron development [38, 39], diacylglycerol kinase beta (*DGKB*) involved in skeletal fragility and stature [40–42], alkylglycerol monooxygenase (*AGMO*) linked to neurological disorder [43], mesenchyme homeobox 2 (*MEOX2*) associated with development of chick embryo [44, 45], isoprenoid synthase domain containing (*ISPD*) related to muscle development of [46, 47]. In addition, phospholipase C beta 4 (*PLCB4*), a gene located on chromosome 3, shows high strong selection signals (Fig. 2D), which is linked to lipid metabolism and sensorimotor experience [48, 49]. Collectively, these results above indicate that *ETV1*, *DGKB*, *AGMO*, *MEOX2*, *ISPD* and *PLCB4* are essential for the neurodevelopment, muscle and skeletal development, fighting performance and sensorimotor functions of gamecocks.

### TreeMix analysis and *D*-statistics revealed gene flows between gamecocks and wild jungle fowls

**Fig. 3:**
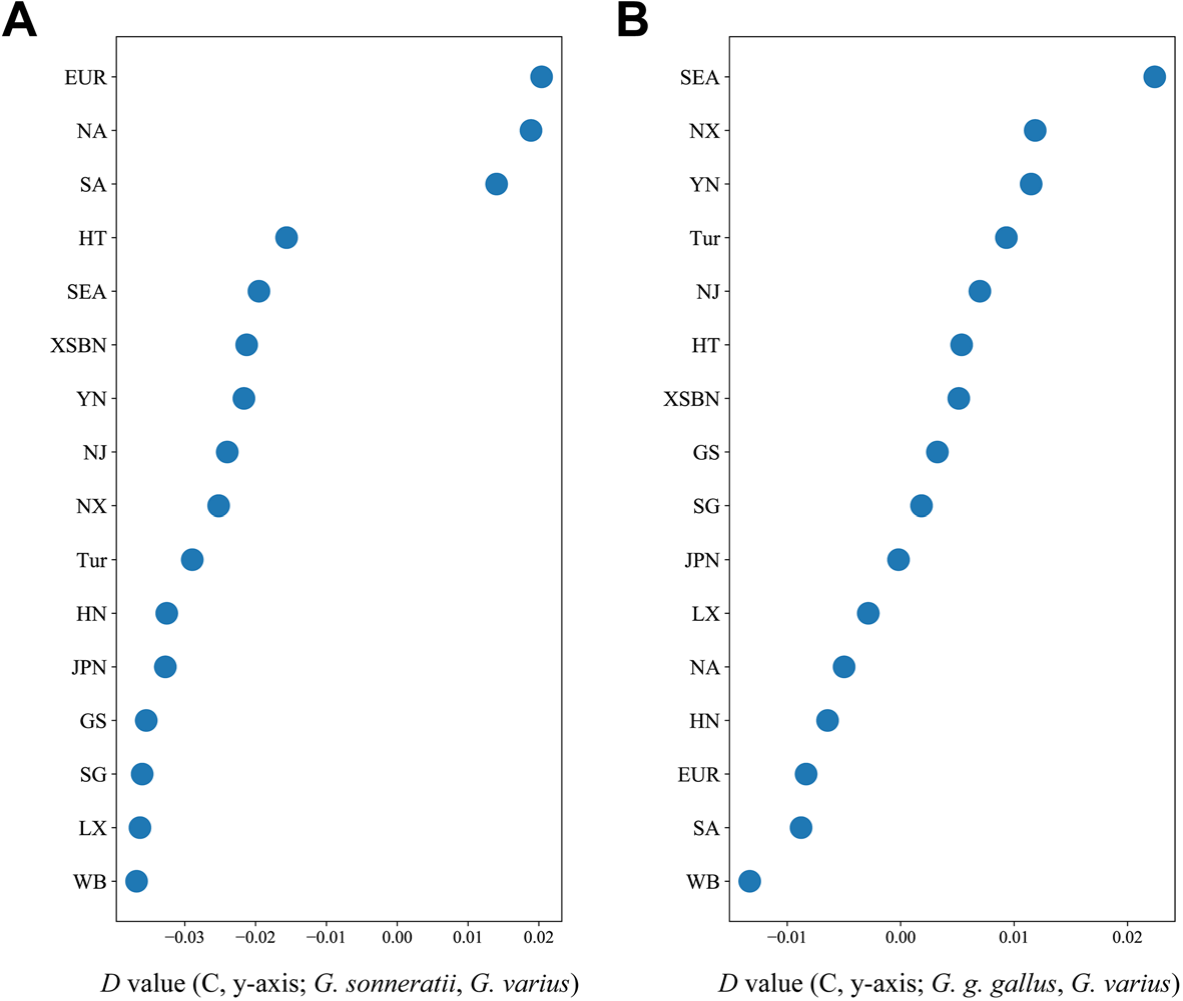
*D*-statistics for gene flow between the wild jungle fowls and gamecocks. The tree topology (P1, P2, P3, O) is as follows: P1 is C, P2 is Target breed (gamecock and Chinese indigenous chicken), P3 is *G. sonneratii* (**A**) or *G. g. gallus* (**B**), and O is *G. varius*

To better understand the historical relationship between gamecocks and other chicken populations, we implemented TreeMix to construct a ML tree with *G. varius* as an outgroup, in which it allows both populations split and migration events. The ML tree showed a similar phylogenetic relationship to the NJ tree above (Fig. 1C) and previous analysis [2]. The ML tree was constructed with migration events given from 0 to 9 (m = 0–9) (Additional file 3: Fig. S2). In the ML tree (m = 9), gene flow was observed between *G. sonneratii* and both EUR and SA gamecock, as well as between *G. g. gallus* and SEA gamecock, indicating potential widespread introgression between wild jungle fowls and gamecocks. In addition, gene flow exists among gamecocks and between gamecocks and C chickens. We further calculated the *D*-statistic value for each gamecock population and other chicken population. The result exhibited possible introgression between *G. sonneratii* and EUR, SA and NA gamecock (Fig. 3A). And another possible introgression event between *G. g. gallus* and SEA gamecock was also detected (Fig. 3B). Besides, we identified (((C, EUR), *G. sonnerattii*), *G. varius*), (((C, NA), *G. sonnerattii*), *G. varius*) and (((C, SA), *G. sonnerattii*), *G. varius*) having the most significant *D* value (Fig. 3A), and the fraction of introgression from the *G. sonnerattii* to EUR, NA and SA gamecock, was 1.47%, 1.40% and 1.05%, respectively (Additional file 1: Table S12). We also found (((C, SEA), *G. g. gallus*), *G. varius*) exhibiting the most significant *D* value (Fig. 3B), indicating a relative high introgression level (4.72%) from *G. g. gallus* to SEA gamecock (Additional file 1: Table S13). Subsequently, we separately studied the genomic regions shared between the *G. sonneratii* and Euramerican gamecock (EUR, SA and NA), as well as between *G. g. gallus* and SEA gamecock.

### Introgression from G. sonneratii to Euramerican gamecock

**Fig. 4:**
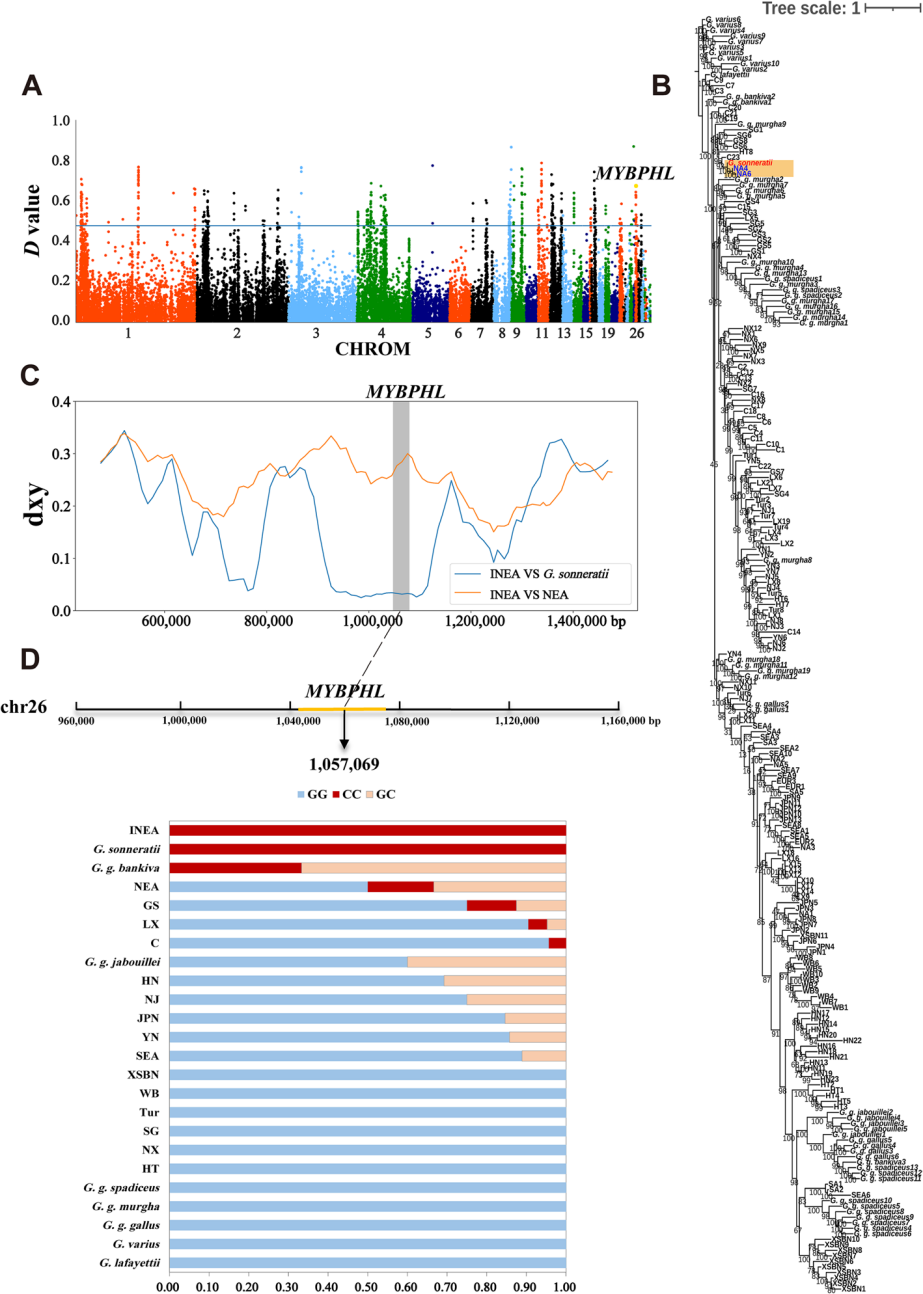
Introgression analysis of *MYBPHL* gene region. **A** Manhattan plot of D values between *G. sonneratii* and Euramerican gamecocks. The blue horizontal line indicates the significance threshold (top 1% of *D* values). **B** The ML tree constructed with the *MYBPHL* gene sequence. The introgression event was highlighted with orange color, the introgressed Euramerican gamecocks and the *G. sonneratii* were highlighted with blue and red color, respectively. **C** The distribution of dxy surrounding the introgressed region in *MYBPHL* between the introgressed Euramerican gamecocks (INEA) and either *G. sonneratii* or remaining non-introgressed Euramerican gamecocks (NEA). **D** Gene structure diagram of *MYBPHL* and allele frequency of mutant *MYBPHL* loci. Blue, red, and orange represent homozygous wild-type, homozygous mutant, and heterozygous mutant, respectively. GGB: *G. g. bankiva*, GGG: *G. g. gallus*, GGM: *G. g. murghi*, GGJ: *G. g. jabouillei*, GGS: *G. g. spadiceus*

Introgression occurs frequently in chickens. Increasing evidence for introgressions between wild jungle fowls and domestic chickens is available [2, 50]. In the study, we detected 181 candidate introgression genes from the *G. sonneratii* to Euramerican gamecock (Fig. 4A, Additional file 1: Table S14–15). Over-representation analysis (corrected *P*-value < 0.05) of GO terms shows that introgressed genes from *G. sonneratii* to Euramerican gamecock were enriched including “glutamatergic synapse”, “postsynaptic density”, “neuropilin binding”, “regulation of skeletal muscle satellite cell proliferation” (Additional file 1: Table S16). KEGG pathways analysis revealed significant overrepresentations (corrected *P*-value < 0.05) of introgressed genes from *G. sonneratii* to Euramerican gamecock was “Autophagy-animal” (Additional file 1: Table S17). Myosin binding protein H (*MYBPH*), as the major component of vertebrate striated muscle fibers, is related to the fibers of rapidly contracting muscles [51]. Taking candidate introgression genes myosin binding protein H-like (*MYBPHL*) as an example, we first constructed an ML tree for the gene region. In the ML tree, we found that NA4 and NA6 clustered with the *G. sonneratii*, while the rest of Euramerican gamecock clustered together with the other chickens (Fig. 4B). Besides, the result of dxy indicated that within the gene region of *MYBPHL*, the dxy between introgressed Euramerican gamecock (NA4 and NA6) and *G. sonneratii* was significantly decreased, while the dxy between introgressed Euramerican gamecock (NA4 and NA6) and other non-introgressed Euramerican chicken was significantly increased (Fig. 4C). What’s more, we also conducted gene variation analysis in the *MYBPHL* region and detected homozygous mutant with a nonsynonymous mutation even fixed in both *G. sonneratii* and the introgressed individual NA4 and NA6, while low frequency or almost absent in other wild jungle fowls, gamecocks, Chinese indigenous chickens and C chickens (Fig. 4D). These observations confirmed that the introgression event at the *MYBPHL* gene region.

### Introgression from *G. g. gallus* to SEA gamecock

**Fig. 5:**
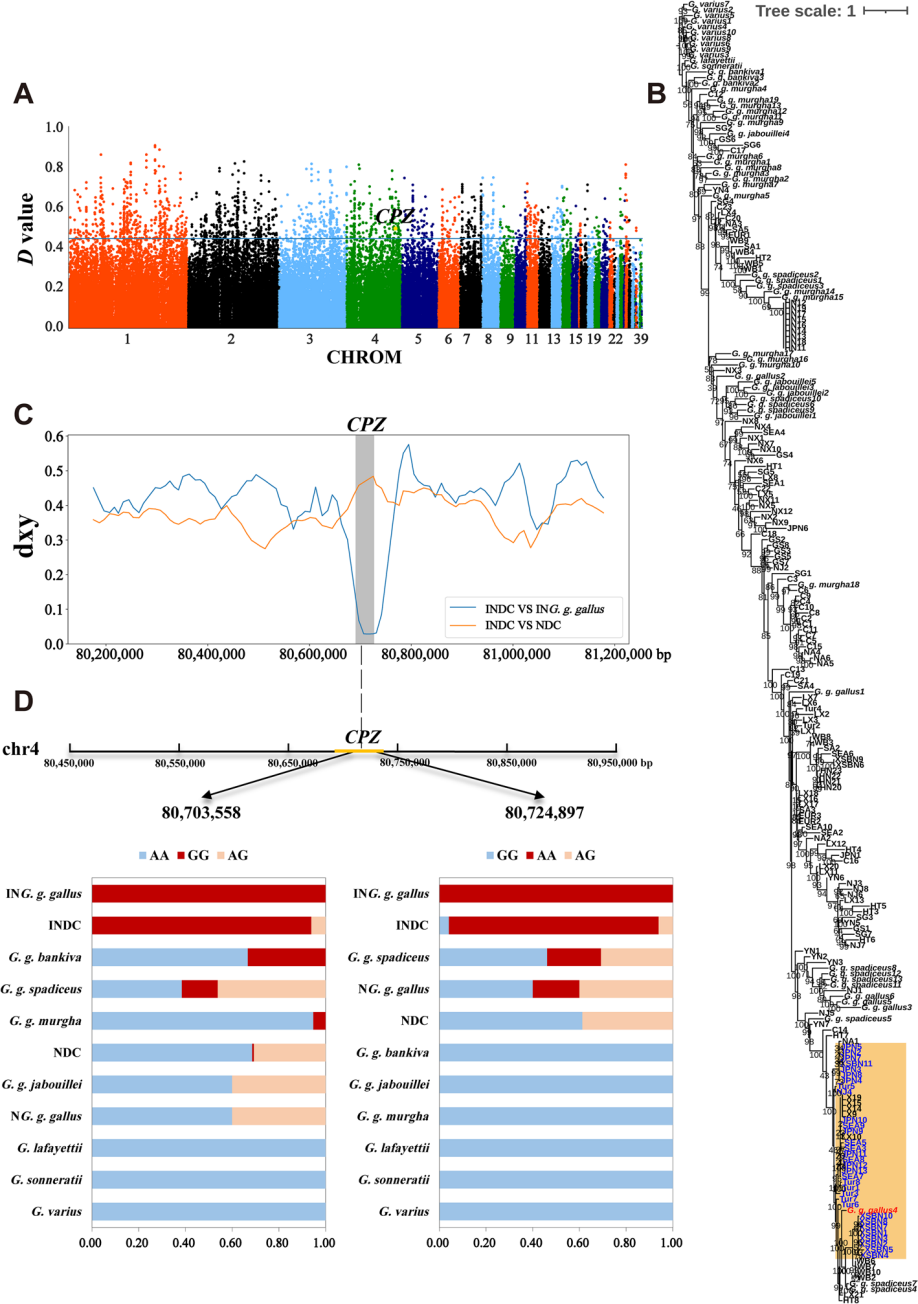
Introgression analysis of *CPZ* gene region. **A** Manhattan plot of D values between *G. g. gallus* and SEA. The blue horizontal line indicates the significance threshold (top 1% of *D* values). **B** The ML tree constructed with the *CPZ* gene sequence. The introgression event was highlighted with orange color, the introgressed SEA and the *G. g. gallus* were highlighted with blue and red color, respectively. **C** The distribution of dxy surrounding the introgressed region in *CPZ* between the introgressed domestic chicken (INDC) and either introgressed *G. g. gallus* (IN*G. g. gallus*) or remaining non-introgressed domestic chicken (NDC). **D** Gene structure diagram of *CPZ* and allele frequency of mutant *CPZ* loci. Blue, red, and orange represent homozygous wild-type, homozygous mutant, and heterozygous mutant, respectively. GGB: *G. g. bankiva*, GGG: *G. g. gallus*, GGM: *G. g. murghi*, GGJ: *G. g. jabouillei*, GGS: *G. g. spadiceus*

We computed the *D*-statistic value across the whole genome in the form *D*-statistic (((C, SEA), *G. g. gallus*), *G. varius*) and identified 436 genes of SEA gamecock exhibited a significant signal of admixture with *G. g. gallus* (Fig. 5A, Additional file 1: Table S18–19). GO analysis revealed significant overrepresentations (corrected *P*-value < 0.05) of introgressed genes from *G. g. gallus* to SEA gamecock were “neuron projection”, “ATP binding”, “glutamatergic synapse”, “synapse”, “startle response”, “axon” and “myosin light chain binding”, and so on (Additional file 1: Table S20). KEGG pathways analysis showed significant overrepresentations (corrected *P*-value < 0.05) of introgressed genes from *G. g. gallus* to SEA gamecock enriched in “Neuroactive ligand-receptor interaction”, “MAPK signaling pathway” and “FoxO signaling pathway” (Additional file 1: Table S21). In particular, we identified several candidate introgression genes related to growth, organism and muscle development, body size as well as weight, such as, *DGKB*, *MEOX2*, insulin like growth factor 1 (*IGF1*), GLI family zinc finger 3 (*GLI3*), calmodulin-lysine N-methyltransferase (*CAMKMT*), cadherin 18 (*CDH18*), decorin (*DCN*), which is also identified by different gamecock breed [17, 45, 52–54]. Candidate introgression gene, brain derived neurotrophic factor (*BDNF*) involve in aggressive behavior [9], and interleukin 1 receptor accessory protein like 1 (*IL1RAPL1*) might be associated with nervous system function [17].

In addition, candidate introgression genes, carboxypeptidase Z (*CPZ*) is likely related to the development of skeletal elements and aggressive behavior [9, 55]. Subsequently, we constructed an ML tree for the gene region of *CPZ*. In the ML tree, we observed that SEA3, SEA5, SEA7, SEA8 and SEA9 cluster with the *G. g. gallus*, while the remaining SEA gamecock clustered together with the other chickens (Fig. 5B). Particularly, in addition to SEA, Tur, XSBN and JPN gamecock also clustered together with *G. g. gallus*. To validate the finding observed from ML tree, we further detected the gene region originating from *G. g. gallus* in the genomes of these gamecock populations (Additional file 1: Table S22–24). The results confirmed that *CPZ* was commonly identified as a candidate introgression gene in these gamecock populations, which support the finding of ML tree. In addition, we noticed a remarkably reduced dxy of the *CPZ* gene region between the introgressed individuals and *G. g. gallus*, in contrast to the significantly increased dxy in the introgressed individuals versus other non-introgressed chickens (Fig. 5C). What’s more, we detected homozygous mutant with two nonsynonymous mutations in the *CPZ* gene region with high frequency or even fixed in both *G. g. gallus* and the introgressed individuals, while low frequency or almost absent in non-introgressed domestic chickens and other wild jungle fowls (Fig. 5D). In conclusion, these results confirmed the introgression event at the *CPZ* gene region.

## Discussion

Advances in sequencing technology have provided the opportunity to fully resolve the origins, domestication, introgression events, and complex traits of domesticated animals. In this study, to explore the origin and unique traits of gamecocks, genetic admixture between gamecock and other chicken breeds, we conducted a comprehensive genomic analysis of whole genome sequence variations for gamecock populations, wild jungle fowls, C chickens, Chinese indigenous chickens. The population structure is essential for genetic assessment, utilization and conservation of gamecock genetic resources. So, we explored the population genetic structure of gamecocks and other chickens. The results of ADMIXTURE analysis, PCA and NJ tree all suggest that gamecocks have multiple origins, which is consistent with the previous finding [8].

Unlike previous studies that only focused on Chinese gamecocks or other regional gamecocks, we identified significant and distinctive signatures of gamecocks from multiple regions worldwide. Combining different selective sweep detection methods can provide complementary information, it is considered to be the optimal strategy for detecting selected signature. Therefore, we mainly focus on the candidate genes identified by *F*st, π-Ratio and XP-EHH. Our results showed that the three methods detected fifteen shared candidate genes. Among these genes, *ETV1*, *DGKB*, *MEOX2, AGMO, ISPD* and *PLCB4* might play an important role in muscular and robust bodies and extremely aggressive behavior in gamecocks. In addition, we also detected several candidate genes of gamecocks, ligand dependent nuclear receptor corepressor like (*LCORL*), insulin like growth factor 2 mRNA binding protein 1 (*IGF2BP1*), ectodysplasin A (*EDA*), CASP2 and RIPK1 domain containing adaptor with death domain (*CRADD*) and suppressor of cytokine signaling 2 (*SOCS2*), ephrin B1 (*EFNB1*), histone deacetylase 9 (*HDAC9*), collagen type VI alpha 1 chain (*COL6A1*), potassium calcium-activated channel subfamily M alpha 1 (*KCNMA1*), glutamate metabotropic receptor 8 (*GRM8*) and *BDNF*, might be related to body size, skeletal, muscle and limb development, and aggressive behavior [7, 9, 45, 52–54]. Gamecocks are ornamental breeds with the basic characteristics of pea-crowned, long neck, tall body, strong and well-developed musculature, especially the leg muscles, in order to adapt to running, jumping, and standing upright, as well as extreme aggression and aggressive behavior. These candidate genes could explain why gamecocks are more muscular and aggressive than other breeds. In addition, cockfighting is a very strenuous and intense form of exercise where the ability to maintain and efficiently distribute metabolism is critical. Candidate genes, agouti related neuropeptide (*AGRP*), gastric inhibitory polypeptide (*GIP*), cyclase associated actin cytoskeleton regulatory protein 1 (*CAP1*), major facilitator superfamily domain containing 2A (*MFSD2A*) are correlated with energy homeostasis, glucose metabolism and cell process [45, 52, 54]. The morphology of the tissues and organs of gamecocks differs from that of other chicken breeds, such as having wider jaw joints and frontal bones [56]. Among these candidate genes, Grainyhead-like transcription factor 3 (*GRHL3*) plays an important role in the growth and development of the craniofacial skeleton, which is consistent with the findings of XSBN fighting chickens [53]. Collectively, we identify these significant candidate genes for skeletal, muscle, limb development and aggressive behavior, which is critical for gamecocks.

Introgression event between different breeds or populations of chickens are a common phenomenon. For example, Indonesian chickens and Chinese chickens possess ancestry from *G. g. bankiva* and *G. g. jabouillei*, respectively, via gene flow [2]. *G. g. murgha* made a substantial genetic contribution to domestic chickens of South Asia via gene flow [2]. Introgression genes contribute significantly to the diversity of chickens at the level of individual or breed-specific variation. Breed-specific introgression may well be related to important economic traits or adaptations. *G. g. murgha* plays a crucial role in the development of C chickens (White Leghorn), contributing 25% of the ancestry [2]. Introgression from *G. sonneratii* may impact the growth trait of domestic chickens [50]. Introgression is widespread in chickens, however, introgression in gamecocks has rarely been studied, specially, introgression event and introgression genes between wild jungle fowl and gamecocks has not been investigated. Here, we found that admixture occurred within gamecock populations and between gamecock and other indigenous chicken populations and between wild jungle fowls and gamecock, consciously or unconsciously. Most importantly, our study providing the first evidence of introgression occurring between wild jungle fowls and gamecocks. Through a series of analysis, including constructing ML tree, detecting dxy and nonsynonymous mutations analysis of *MYBPHL* and *CPZ* gene region, we confirmed the introgression events at the *MYBPHL* and *CPZ* gene region from the wild jungle fowls into gamecock populations. However, the exact function of the two introgressed genes are still unclear. Thus, it is necessary to conduct phenotyping experiments between the introgressed individuals and the non-introgressed gamecocks to probe deeper into the effects of introgression.

Through whole genome sequencing data of large-scale gamecock individuals, our study confirmed the origin of gamecock and extended our understanding of introgression event of gamecocks, highlighting the crucial role played in chicken breeding and also laying a theoretical foundation for future research of gamecocks.

## Conclusion

This study provided a comprehensive overview of gamecocks from multiple regions worldwide through whole genome sequencing. Our analysis reveals that gamecocks have multiple origins and identifies several candidate genes that may play critical roles in muscle development, body formation, nervous system function, and fighting performance—key factors for gamecocks breeding and development. More importantly, our study is the first to detect introgression event and candidate introgressed genes from wild jungle fowls to gamecocks. Our findings are significant for the conservation and utilization of gamecocks, providing a theoretical basis for understanding muscle development mechanism and behavior pattern in chickens, as well as offering reliable genetic resources for the genetic improvement of domestic chickens.

## Supplementary Information

Additional file 1: Table S1. Summary of all sample data. Table S2. The candidate genes of top 1% *F*st. Table S3. The candidate genes of top 1% π-Ratio. Table S4. The candidate genes of top 1% XP-EHH. Table S5. Summary information of candidate genes shared by three methods and other focused candidate genes. Table S6. GO enrichment analysis on the candidate gene identified by top 1% *F*st. Table S7. GO enrichment analysis on the candidate gene identified by top 1% π-Ratio. Table S8. GO enrichment analysis on the candidate gene identified by top 1% XP-EHH. Table S9. KEGG enrichment analysis on the candidate gene identified by top 1% *F*st. Table S10. KEGG enrichment analysis on the candidate gene identified by top 1% π-Ratio. Table S11. KEGG enrichment analysis on the candidate gene identified by top 1% XP-EHH. Table S12. Results of *D*- statistics in the form, *G. sonnerattii*), *G. varius*). Table S13. Results of *D*-statistics in the form, *G. g. gallus*), *G. varius*). Table S14. Significant introgression region between *G. sonnerattii* and Euramerican gamecocks. Table S15. Candidate introgression genes between *G. sonnerattii* and Euramerican gamecocks. Table S16. GO enrichment analysis on the candidate introgression genes between *G. sonnerattii *and Euramerican gamecocks. Table S17. KEGG enrichment analysis on the candidate introgression genes between *G. sonnerattii *and Euramerican gamecocks. Table S18. Significant introgression region between *G. g. gallus* and SEA. Table S19. Candidate introgression genes between *G. g. gallus* and SEA. Table S20. Go enrichment analysis on candidate introgression genes between *G. g. gallus* and SEA. Table S21. KEGG enrichment analysis on the candidate introgression genes between *G. g. gallus* and SEA. Table S22. Candidate introgression genes between *G. g. gallus* and Tur. Table S23. Candidate introgression genes between *G. g. gallus* and XSBN. Table S24. Candidate introgression genes between *G. g. gallus* and JPN.

Additional file 2: Fig. S1. Outgroup f3 statistics, with higher f3 values suggesting more ancestral alleles shared by gamecock and X, and thus their closer relationship.

Additional file 3: Fig. S2. TreeMix analysis of all populations of the study. Migration edges ranging from 0–9 were shown here. The migration weight is represented by the color of the arrow. The scale bar represents 10 times the average standard error of the entries in the sample covariance matrix.

## Data Availability

Raw FASTQ sequences have been deposited to NCBI Short Read Archive under the BioProject accession number PRJNA1158212 (data available after 30 June 2025).

## Abbreviations

AGMO: Alkylglycerol monooxygenase
AGRP: Agouti related neuropeptide
BDNF: Brain derived neurotrophic factor
C chicken: Commercial chicken
CAMKMT: Calmodulin-lysine N-methyltransferase
CAP1: Cyclase associated actin cytoskeleton regulatory protein 1
CDH18: Cadherin 18
COL6A1: Collagen type VI alpha 1 chain
CPZ: Carboxypeptidase Z
CRADD: CASP2 and RIPK1 domain containing adaptor with death domain
DCN: Decorin
DGKB: Diacylglycerol kinase beta
dxy: Mean pairwise sequence divergence
EDA: Ectodysplasin A
EFNB1: Ephrin B1
ETV1: ETS variant 1
EUR gamecock: Europe gamecock
*F*st: Population differentiation index
*G*. *lafayettii*: Ceylon jungle fowl
*G*. *sonneratii*: Grey jungle fowl
*G*. *varius*: Green jungle fowl
GIP: Gastric inhibitory polypeptide
GLI3: GLI family zinc finger 3
GRHL3: Grainyhead-like transcription factor 3
GRM8: Glutamate metabotropic receptor 8
GS chicken: Gushi chicken
HDAC9: Histone deacetylase 9
HN gamecock: Henan gamecock
HT chicken: Hetian chicken
IGF1: Insulin like growth factor 1
IGF2BP1: Insulin like growth factor 2 mRNA binding protein 1
IL1RAPL1: Interleukin 1 receptor accessory protein like 1
ISPD: Isoprenoid synthase domain containing
JPN gamecock: Japanese gamecock
KCNMA1: Potassium calcium-activated channel subfamily M alpha 1
LCORL: Ligand dependent nuclear receptor corepressor like
LX gamecock: Luxi gamecock
MEOX2: Mesenchyme homeobox 2
MFSD2A: Major facilitator superfamily domain containing 2A
MYBPH: Myosin binding protein H
MYBPHL: Myosin binding protein H-like
NA gamecock: North America gamecock
NJ gamecock: Nanjiang gamecock
NX chicken: Nixi chicken
PCA: Principal component analysis
PLCB4: Phospholipase C beta 4
RJF: Red jungle fowl
SA gamecock: South America gamecock
SEA gamecock: Southeast Asia gamecock
SG chicken: Shouguang chicken
SNPs: Single nucleotide polymorphisms
SOCS2: Suppressor of cytokine signaling 2
Tur gamecock: Turpan gamecock
WB gamecock: Wanbei gamecock
XP-EHH: Cross-population extended haplotype homozygosity
XSBN gamecock: Xishuangbanna gamecock
YN gamecock: Yunnan gamecock
π-Ratio: The largest differences in genetic diversity

## References

[1] Food and Agriculture Organization of the United Nations. Gateway to poultry production and products: chickens. 2023. https://www.fao.org/poultry-production-products/production/poultry-species/chickens/en/. Accessed 2023.

[2] Wang MS, Thakur M, Peng MS, Jiang Y, Frantz LAF, Li M, et al. 863 genomes reveal the origin and domestication of chicken. Cell Res. 2020;30(8):693–701. 32581344 10.1038/s41422-020-0349-y PMC7395088

[3] Ren X, Guan Z, Li H, Zhang L, Wen J, Zhao X, et al. Phylogenetic analysis reveals multiple origins of Chinese gamecocks. Poult Sci. 2023;102(12):103068. 37778296 10.1016/j.psj.2023.103068 PMC10550403

[4] Bendesky A, Brew J, Francis KX, Tello Corbetto EF, González Ariza A, Nogales Baena S, et al. The main genetic locus associated with the evolution of gamecocks is centered on ISPD. G3 (Bethesda). 2024;14(2):jkad267. 10.1093/g3journal/jkad267 PMC10849328 37991999

[5] Mahmood S, Khan MS, Ullah Z, Lawal RA, Hanotte O. The origin of genetic diversity of indigenous cockfighting chickens of Pakistan by analyzing the mtDNA. Heliyon. 2024;10(6):e27755. 38545210 10.1016/j.heliyon.2024.e27755 PMC10965519

[6] Endo H, Mori K, Hosojima M, Mekwichai W, Ogawa H, Tsunekawa N, et al. Functional-morphological characteristics in the musculoskeletal system of standing-type cocks including some game breeds. Jpn J Zoo Wildl Med. 2012;17:131–8.

[7] Bi H, Yi G, Yang N. Increased copy number of SOCS2 gene in Chinese gamecocks. Poult Sci. 2017;96(5):1041–4. 28008131 10.3382/ps/pew391

[8] Ren X, Guan Z, Li H, Wen J, Zhao X, Wang G, et al. Extensive intra- and inter-genetic admixture of Chinese gamecock and other indigenous chicken breeds revealed by genomic data. Poult Sci. 2023;102(7):102766. 37229885 10.1016/j.psj.2023.102766 PMC10220482

[9] Luo W, Luo C, Wang M, Guo L, Chen X, Li Z, et al. Genome diversity of Chinese indigenous chicken and the selective signatures in Chinese gamecock chicken. Sci Rep. 2020;10:14532. 32883984 10.1038/s41598-020-71421-z PMC7471287

[10] Sabeti PC, Schaffner SF, Fry B, Lohmueller J, Varilly P, Shamovsky O, et al. Positive natural selection in the human lineage. Science. 2006;312(5780):1614–20. 16778047 10.1126/science.1124309

[11] Ai H, Fang X, Yang B, Huang Z, Chen H, Mao L, et al. Adaptation and possible ancient interspecies introgression in pigs identified by whole-genome sequencing. Nat Genet. 2015;47(3):217–25. 25621459 10.1038/ng.3199

[12] Xu NY, Si W, Li M, Gong M, Larivière JM, Nanaei HA, et al. Genome-wide scan for selective footprints and genes related to cold tolerance in Chantecler chickens. Zool Res. 2021;42(6):710–20. 34581031 10.24272/j.issn.2095-8137.2021.189 PMC8645888

[13] Zheng Z, Wang X, Li M, Li Y, Yang Z, Wang X, et al. The origin of domestication genes in goats. Sci Adv. 2020;6(21):eaaz5216. 10.1126/sciadv.aaz5216 PMC7314551 32671210

[14] Chen N, Xia X, Hanif Q, Zhang F, Dang R, Huang B, et al. Global genetic diversity, introgression, and evolutionary adaptation of indicine cattle revealed by whole genome sequencing. Nat Commun. 2023;14:7803. 38016956 10.1038/s41467-023-43626-z PMC10684552

[15] Ulfah M, Kawahara-Miki R, Farajalllah A, Muladno M, Dorshorst B, Martin A, et al. Genetic features of red and green junglefowls and relationship with Indonesian native chickens Sumatera and Kedu Hitam. BMC Genomics. 2016;17:320. 27142387 10.1186/s12864-016-2652-z PMC4855759

[16] Zhong HA, Kong XY, Zhang YW, Su YK, Zhang B, Zhu L, et al. Microevolutionary mechanism of high-altitude adaptation in Tibetan chicken populations from an elevation gradient. Evol Appl. 2022;15(12):2100–12. 36540645 10.1111/eva.13503 PMC9753841

[17] Zhou J, Chang Y, Li J, Bao H, Wu C. Integrating whole-genome resequencing and RNA sequencing data reveals selective sweeps and differentially expressed genes related to nervous system changes in Luxi gamecocks. Genes (Basel). 2023;14(3):584. 36980855 10.3390/genes14030584 PMC10048732

[18] Bolger AM, Lohse M, Usadel B. Trimmomatic: a flexible trimmer for Illumina sequence data. Bioinformatics. 2014;30:2114–20. 24695404 10.1093/bioinformatics/btu170 PMC4103590

[19] Abuín JM, Pichel JC, Pena TF, Amigo J. BigBWA: approaching the Burrows-Wheeler aligner to Big Data technologies. Bioinformatics. 2015;31:4003–5. 26323715 10.1093/bioinformatics/btv506

[20] Li H, Handsaker B, Wysoker A, Fennell T, Ruan J, Homer N, et al. The sequence alignment/map format and SAMtools. Bioinformatics. 2009;25:2078–9. 19505943 10.1093/bioinformatics/btp352 PMC2723002

[21] McKenna A, Hanna M, Banks E, Sivachenko A, Cibulskis K, Kernytsky A, et al. The Genome Analysis Toolkit: a MapReduce framework for analyzing next-generation DNA sequencing data. Genome Res. 2010;20:1297–1303. 10.1101/gr.107524.110 PMC2928508 20644199

[22] Browning SR, Browning BL. Rapid and accurate haplotype phasing and missing-data inference for whole-genome association studies by use of localized haplotype clustering. Am J Hum Genet. 2007;81:1084–97. 17924348 10.1086/521987 PMC2265661

[23] Letunic I, Bork P. Interactive Tree Of Life (iTOL) v4: recent updates and new developments. Nucleic Acids Res. 2019;47(W1):W256–9. 30931475 10.1093/nar/gkz239 PMC6602468

[24] Danecek P, Auton A, Abecasis G, Albers CA, Banks E, DePristo MA, et al. The variant call format and VCFtools. Bioinformatics. 2011;27:2156–8. 21653522 10.1093/bioinformatics/btr330 PMC3137218

[25] Cingolani P, Platts A, le Wang L, Coon M, Nguyen T, Wang L, et al. A program for annotating and predicting the effects of single nucleotide polymorphisms, SnpEff: SNPs in the genome of *Drosophila melanogaster* strain w^1118^; iso-2; iso-3. Fly (Austin). 2012;6(2):80–92. 10.4161/fly.19695 PMC3679285 22728672

[26] Patterson N, Price AL, Reich D. Population structure and eigenanalysis. PLoS Genet. 2006;2(12):e190. 17194218 10.1371/journal.pgen.0020190 PMC1713260

[27] Alexander DH, Novembre J, Lange K. Fast model-based estimation of ancestry in unrelated individuals. Genome Res. 2009;19(9):1655–64. 19648217 10.1101/gr.094052.109 PMC2752134

[28] Patterson N, Moorjani P, Luo Y, Mallick S, Rohland N, Zhan Y, et al. Ancient admixture in human history. Genetics. 2012;192:1065–93. 22960212 10.1534/genetics.112.145037 PMC3522152

[29] Weir BS, Cockerham CC. Estimating F-statistics for the analysis of population structure. Evolution. 1984;38(6):1358–70. 28563791 10.1111/j.1558-5646.1984.tb05657.x

[30] Sabeti PC, Varilly P, Fry B, Lohmueller J, Hostetter E, Cotsapas C, et al. Genome-wide detection and characterization of positive selection in human populations. Nature. 2007;449:913–8. 17943131 10.1038/nature06250 PMC2687721

[31] Tang K, Thornton KR, Stoneking M. A new approach for using genome scans to detect recent positive selection in the human genome. PLoS Biol. 2007;5:e171. 17579516 10.1371/journal.pbio.0050171 PMC1892573

[32] Szpiech ZA, Hernandez RD. Selscan: an efficient multithreaded program to perform EHH-based scans for positive selection. Mol Biol Evol. 2014;31(10):2824–7. 25015648 10.1093/molbev/msu211 PMC4166924

[33] Xie C, Mao X, Huang J, Ding Y, Wu J, Dong S, et al. KOBAS 2.0: a web server for annotation and identification of enriched pathways and diseases. Nucleic Acids Res. 2011;39(Web Server issue):W316–22. 10.1093/nar/gkr483 PMC3125809 21715386

[34] Pickrell JK, Pritchard JK. Inference of population splits and mixtures from genome-wide allele frequency data. PLoS Genet. 2012;8:e1002967. 23166502 10.1371/journal.pgen.1002967 PMC3499260

[35] Malinsky M, Matschiner M, Svardal H. Dsuite-Fast D-statistics and related admixture evidence from VCF files. Mol Ecol Resour. 2021;21:584–95. 33012121 10.1111/1755-0998.13265 PMC7116594

[36] Nguyen LT, Schmidt HA, von Haeseler A, Minh BQ. Iq-tree: A fast and effective stochastic algorithm for estimating maximum-likelihood phylogenies. Mol Biol Evol. 2015;32(1):268–74. 25371430 10.1093/molbev/msu300 PMC4271533

[37] Martin SH, Davey JW, Jiggins CD. Evaluating the use of ABBA-BABA statistics to locate introgressed loci. Mol Biol Evol. 2015;32(1):244–57. 25246699 10.1093/molbev/msu269 PMC4271521

[38] Tenney AP, Livet J, Belton T, Prochazkova M, Pearson EM, Whitman MC, et al. Etv1 controls the establishment of non-overlapping motor innervation of neighboring facial muscles during development. Cell Rep. 2019;29(2):437–52. 31597102 10.1016/j.celrep.2019.08.078 PMC7032945

[39] de Nooij JC, Doobar S, Jessell TM. Etv1 inactivation reveals proprioceptor subclasses that reflect the level of NT3 expression in muscle targets. Neuron. 2013;77(6):1055–68. 23522042 10.1016/j.neuron.2013.01.015 PMC3763960

[40] Costantini A, Skarp S, Kämpe A, Mäkitie RE, Pettersson M, Männikkö M, et al. Rare copy number variants in array-based comparative genomic hybridization in early-onset skeletal fragility. Front Endocrinol (Lausanne). 2018;9:380. 30042735 10.3389/fendo.2018.00380 PMC6048219

[41] Dupuis J, Langenberg C, Prokopenko I, Saxena R, Soranzo N, Jackson AU, et al. New genetic loci implicated in fasting glucose homeostasis and their impact on type 2 diabetes risk. Nat Genet. 2010;42(2):105–16. 20081858 10.1038/ng.520 PMC3018764

[42] Wit JM, van Duyvenvoorde HA, van Klinken JB, Caliebe J, Bosch CA, Lui JC, et al. Copy number variants in short children born small for gestational age. Horm Res Paediatr. 2014;82(5):310–8. 25300501 10.1159/000367712 PMC4236248

[43] Alrayes N, Mohamoud HS, Ahmed S, Almramhi MM, Shuaib TM, Wang J, et al. The alkylglycerol monooxygenase (AGMO) gene previously involved in autism also causes a novel syndromic form of primary microcephaly in a consanguineous Saudi family. J Neurol Sci. 2016;363:240–4. 27000257 10.1016/j.jns.2016.02.063

[44] Reijntjes S, Stricker S, Mankoo BS. A comparative analysis of Meox1 and Meox2 in the developing somites and limbs of the chick embryo. Int J Dev Biol. 2007;51(8):753–9. 17939123 10.1387/ijdb.072332sr

[45] Ren X, Guan Z, Zhao X, Zhang X, Wen J, Cheng H, et al. Systematic selection signature analysis of Chinese gamecocks based on genomic and transcriptomic data. Int J Mol Sci. 2023;24(6):5868. 36982941 10.3390/ijms24065868 PMC10059269

[46] Cirak S, Foley AR, Herrmann R, Willer T, Yau S, Stevens E, et al. ISPD gene mutations are a common cause of congenital and limb-girdle muscular dystrophies. Brain. 2013;136:269–81. 23288328 10.1093/brain/aws312 PMC3562076

[47] Guo L, Zhang S, Xu Y, Huang Y, Luo W, Wen Q, et al. A missense mutation in ISPD contributes to maintain muscle fiber stability. Poult Sci. 2022;101(11):102143. 36167018 10.1016/j.psj.2022.102143 PMC9513258

[48] Lee CW, Park DJ, Lee KH, Kim CG, Rhee SG. Purification, molecular cloning, and sequencing of phospholipase C-beta 4. J Biol Chem. 1993;268(28):21318–27. 8407970

[49] Chen X, Du Y, Broussard GJ, Kislin M, Yuede CM, Zhang S, et al. Transcriptomic mapping uncovers Purkinje neuron plasticity driving learning. Nature. 2022;605(7911):722–7. 35545673 10.1038/s41586-022-04711-3 PMC9887520

[50] Zhao X, Wen J, Zhang X, Zhang J, Zhu T, Wang H, et al. Significant genomic introgression from grey junglefowl (*Gallus sonneratii*) to domestic chickens (*Gallus gallus domesticus*). J Anim Sci Biotechnol. 2024;15:45. 38556896 10.1186/s40104-024-01006-7 PMC10983685

[51] Gilbert R, Cohen JA, Pardo S, Basu A, Fischman DA. Identification of the A-band localization domain of myosin binding proteins C and H (MyBP-C, MyBP-H) in skeletal muscle. J Cell Sci. 1999;112(Pt 1):69–79. 9841905 10.1242/jcs.112.1.69

[52] Zhi Y, Wang D, Zhang K, Wang Y, Geng W, Chen B, et al. Genome-wide genetic structure of Henan indigenous chicken breeds. Animals (Basel). 2023;13(4):753. 36830540 10.3390/ani13040753 PMC9952073

[53] Guo X, Fang Q, Ma C, Zhou B, Wan Y, Jiang R. Whole-genome resequencing of Xishuangbanna fighting chicken to identify signatures of selection. Genet Sel Evol. 2016;48:62. 27565441 10.1186/s12711-016-0239-4 PMC5000499

[54] Rostamzadeh Mahdabi E, Esmailizadeh A, Ayatollahi Mehrgardi A, Asadi FM. A genome-wide scan to identify signatures of selection in two Iranian indigenous chicken ecotypes. Genet Sel Evol. 2021;53:72. 34503452 10.1186/s12711-021-00664-9 PMC8428137

[55] Moeller C, Swindell EC, Kispert A, Eichele G. Carboxypeptidase Z (CPZ) modulates Wnt signaling and regulates the development of skeletal elements in the chicken. Development. 2003;130(21):5103–11. 12944424 10.1242/dev.00686

[56] Kudo K, Tsunekawa N, Ogawa H, Endo H. Comparative functional morphology of skulls among Japanese breeds of domestic fowls. J Poult Sci. 2016;53:43–50. 32908363 10.2141/jpsa.0150055 PMC7477248

