# Supplementary figures and images for: Population structure, selection signal and introgression of gamecocks revealed by whole genome sequencing
Source: J Anim Sci Biotechnol. 2025 Feb 8;16:22. doi: 10.1186/s40104-025-01154-4 (PMC11806877; doi:10.1186/s40104-025-01154-4)

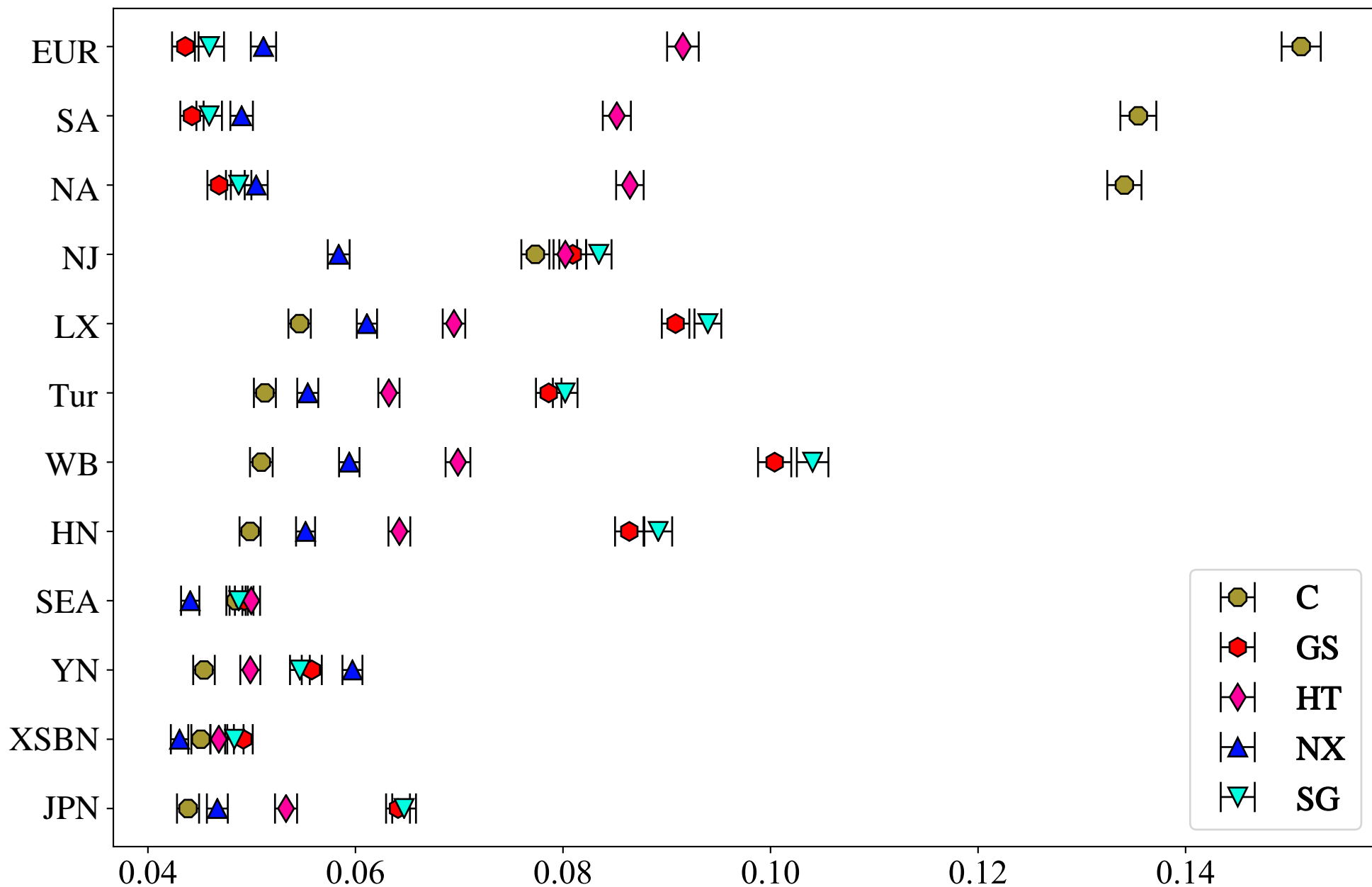

Outgroup  $f_3(X, \text{Gamecock}; G. g. \text{spadiceus})$

Supplement: Supplementary file 2 — Additional file 2: Fig. S1. Outgroup f3 statistics, with higher f3 values suggesting more ancestral alleles shared by gamecock and X, and thus their closer relationship. [file 40104_2025_1154_MOESM2_ESM.pdf]
