# Supplementary material for: Population structure, selection signal and introgression of gamecocks revealed by whole genome sequencing
Source: J Anim Sci Biotechnol. 2025 Feb 8;16:22. doi: 10.1186/s40104-025-01154-4 (PMC11806877; doi:10.1186/s40104-025-01154-4)
Supplement: Supplementary file 3 — Additional file 3: Fig. S2. TreeMix analysis of all populations of the study. Migration edges ranging from 0–9 were shown here. The migration weight is represented by the color of the arrow. The scale bar represents 10 times the average standard error of the entries in the sample covariance matrix. [file 40104_2025_1154_MOESM3_ESM.pdf]

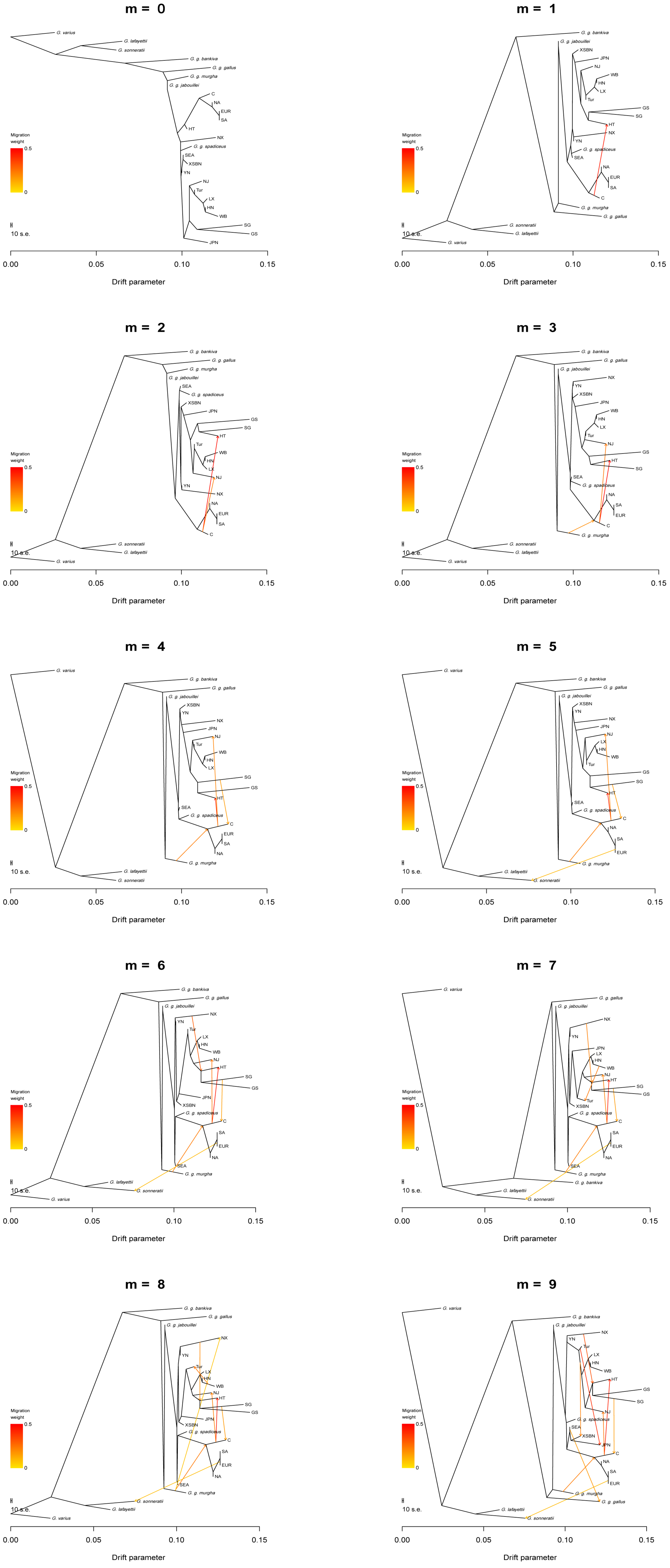
m = 0
G. varius
G. lafayettii
G. sonneratii
G. g. bankiva
G. g. gallus
G. g. murgha
G. g. jabouillei
C
NA
EUR
SA
HT
NX
G. g. spadiceus
SEA
XSBN
YN
NJ
Tur
LX
HN
WB
SG
GS
JPN
Migration weight
0.5
0
10 s.e.
0.00
0.05
0.10
0.15
Drift parameter
m = 1
G. g. bankiva
G. g. jabouillei
XSBN
JPN
NJ
WB
HN
LX
Tur
GS
SG
HT
NX
YN
G. g. spadiceus
SEA
NA
EUR
SA
C
G. g. murgha
G. g. gallus
G. sonneratii
G. lafayettii
G. varius
Migration weight
0.5
0
10 s.e.
0.00
0.05
0.10
0.15
Drift parameter
m = 2
G. g. bankiva
G. g. gallus
G. g. murgha
G. g. jabouillei
SEA
G. g. spadiceus
XSBN
JPN
GS
SG
HT
Tur
WB
HN
LX
NJ
YN
NX
NA
EUR
SA
C
G. sonneratii
G. lafayettii
G. varius
Migration weight
0.5
0
10 s.e.
0.00
0.05
0.10
0.15
Drift parameter
m = 3
G. g. bankiva
G. g. gallus
G. g. jabouillei
NX
YN
XSBN
JPN
WB
HN
LX
Tur
NJ
GS
HT
SG
SEA
G. g. spadiceus
NA
SA
EUR
C
G. g. murgha
G. sonneratii
G. lafayettii
G. varius
Migration weight
0.5
0
10 s.e.
0.00
0.05
0.10
0.15
Drift parameter
m = 4
G. varius
G. g. bankiva
G. g. gallus
G. g. jabouillei
XSBN
YN
NX
JPN
NJ
Tur
WB
HN
LX
SG
GS
HT
SEA
G. g. spadiceus
C
EUR
SA
NA
G. g. murgha
G. lafayettii
G. sonneratii
Migration weight
0.5
0
10 s.e.
0.00
0.05
0.10
0.15
Drift parameter
m = 5
G. varius
G. g. bankiva
G. g. gallus
G. g. jabouillei
XSBN
YN
NX
JPN
NJ
LX
HN
WB
Tur
GS
SG
HT
SEA
G. g. spadiceus
C
NA
SA
EUR
G. g. murgha
G. lafayettii
G. sonneratii
Migration weight
0.5
0
10 s.e.
0.00
0.05
0.10
0.15
Drift parameter
m = 6
G. g. bankiva
G. g. gallus
G. g. jabouillei
NX
YN
Tur
LX
HN
WB
NJ
HT
SG
GS
JPN
XSBN
G. g. spadiceus
C
SA
EUR
NA
SEA
G. g. murgha
G. lafayettii
G. sonneratii
G. varius
Migration weight
0.5
0
10 s.e.
0.00
0.05
0.10
0.15
Drift parameter
m = 7
G. varius
G. g. gallus
G. g. jabouillei
NX
YN
JPN
LX
HN
WB
NJ
HT
SG
GS
Tur
XSBN
G. g. spadiceus
C
SA
EUR
NA
SEA
G. g. murgha
G. g. bankiva
G. lafayettii
G. sonneratii
Migration weight
0.5
0
10 s.e.
0.00
0.05
0.10
0.15
Drift parameter
m = 8
G. g. bankiva
G. g. gallus
G. g. jabouillei
NX
YN
Tur
LX
HN
WB
NJ
HT
SG
GS
JPN
XSBN
G. g. spadiceus
C
SA
EUR
NA
SEA
G. g. murgha
G. sonneratii
G. lafayettii
G. varius
Migration weight
0.5
0
10 s.e.
0.00
0.05
0.10
0.15
Drift parameter
m = 9
G. varius
G. g. bankiva
G. g. jabouillei
NX
YN
Tur
LX
HN
WB
HT
GS
SG
NJ
G. g. spadiceus
SEA
XSBN
JPN
C
NA
SA
EUR
G. g. murgha
G. g. gallus
G. lafayettii
G. sonneratii
Migration weight
0.5
0
10 s.e.
0.00
0.05
0.10
0.15
Drift parameter
